# Scrutiny of chimeric antigen receptor activation by the extracellular domain: experience with single domain antibodies targeting multiple myeloma cells highlights the need for case-by-case optimization

**DOI:** 10.3389/fimmu.2024.1389018

**Published:** 2024-04-19

**Authors:** Heleen Hanssens, Fien Meeus, Yannick De Vlaeminck, Quentin Lecocq, Janik Puttemans, Pieterjan Debie, Timo W. M. De Groof, Cleo Goyvaerts, Kim De Veirman, Karine Breckpot, Nick Devoogdt

**Affiliations:** ^1^ Laboratory of Molecular Imaging and Therapy (MITH), Department of Biomedical Sciences, Vrije Universiteit Brussel, Brussels, Belgium; ^2^ Laboratory for Molecular and Cellular Therapy (LMCT), Translational Oncology Research Center, Department of Biomedical Sciences, Vrije Universiteit Brussel, Brussels, Belgium; ^3^ Laboratory for Hematology and Immunology (HEIM), Translational Oncology Research Center, Department of Biomedical Sciences, Vrije Universiteit Brussel, Brussels, Belgium

**Keywords:** CAR-T cells, VHH, multiple myeloma, adoptive cell transfer, hematology

## Abstract

**Introduction:**

Multiple myeloma (MM) remains incurable, despite the advent of chimeric antigen receptor (CAR)-T cell therapy. This unfulfilled potential can be attributed to two untackled issues: the lack of suitable CAR targets and formats. In relation to the former, the target should be highly expressed and reluctant to shedding; two characteristics that are attributed to the CS1-antigen. Furthermore, conventional CARs rely on scFvs for antigen recognition, yet this withholds disadvantages, mainly caused by the intrinsic instability of this format. VHHs have been proposed as valid scFv alternatives. We therefore intended to develop VHH-based CAR-T cells, targeting CS1, and to identify VHHs that induce optimal CAR-T cell activation together with the VHH parameters required to achieve this.

**Methods:**

CS1-specific VHHs were generated, identified and fully characterized, *in vitro* and *in vivo*. Next, they were incorporated into second-generation CARs that only differ in their antigen-binding moiety. Reporter T-cell lines were lentivirally transduced with the different VHH-CARs and CAR-T cell activation kinetics were evaluated side-by-side. Affinity, cell-binding capacity, epitope location, *in vivo* behavior, binding distance, and orientation of the CAR-T:MM cell interaction pair were investigated as predictive parameters for CAR-T cell activation.

**Results:**

Our data show that the VHHs affinity for its target antigen is relatively predictive for its *in vivo* tumor-tracing capacity, as tumor uptake generally decreased with decreasing affinity in an *in vivo* model of MM. This does not hold true for their CAR-T cell activation potential, as some intermediate affinity-binding VHHs proved surprisingly potent, while some higher affinity VHHs failed to induce equal levels of T-cell activation. This could not be attributed to cell-binding capacity, *in vivo* VHH behavior, epitope location, cell-to-cell distance or binding orientation. Hence, none of the investigated parameters proved to have significant predictive value for the extent of CAR-T cell activation.

**Conclusions:**

We gained insight into the predictive parameters of VHHs in the CAR-context using a VHH library against CS1, a highly relevant MM antigen. As none of the studied VHH parameters had predictive value, defining VHHs for optimal CAR-T cell activation remains bound to serendipity. These findings highlight the importance of screening multiple candidates.

## 1 Introduction

Multiple Myeloma (MM) is a cancer of mature, antibody-producing B cells (plasma cells) that grow uncontrollably in the bone marrow. They thereby disturb the bone formation process and hematopoietic equilibrium, leading to characteristic bone lesions, hypercalcemia and general anemia (1). They also produce excessive amounts of dysfunctional immunoglobulin molecules (M- or paraprotein), causing renal problems (2). Worldwide, MM accounts for 14% of all hematological cancers, making it the third most observed one. It is considered a treatable but incurable malignancy, with a 5-year overall survival rate of 54% (3).

Standard induction therapy for newly diagnosed MM patients usually comprises a combination of immunomodulatory drugs (e.g., lenalidomide) and corticosteroids (e.g., dexamethasone), most often combined with proteasome inhibitors (e.g., bortezomib) (1, 4). This regimen is supplemented with anti-CD38 monoclonal antibody (mAb)-therapy (daratumumab) in some cases. For all eligible patients, induction therapy is followed by autologous stem cell transplantation (SCT). Subsequent maintenance therapy may be bortezomib- or lenalidomide-based (4). Although these regimens are often initially successful, relapse with an increased tolerance to previous treatment regimens is a commonly observed phenomenon. Upon relapse, combination treatment options are diverse and adjusted to the patient specifically (2).

It is established that the MM tumor microenvironment is highly immunosuppressive, among others due to disturbed cytokine production by malignant, stromal and immune cell populations and an outbalanced programmed death-1 (PD-1)/PD-1 ligand (PD-L1) immune checkpoint axis (5). This leads to a malfunctioning innate and adaptive immune environment, involving both myeloid and lymphoid actors. Increased understanding about these aberrances, together with observed graft-versus-myeloma effects in early allogenic SCT trials, highlight the potential added value of immune therapy for MM (6). Forms of adoptive cell transfer currently being evaluated for the treatment of MM include T-cell receptor (TCR)-modified T-cells, (allogenic) chimeric antigen receptor (CAR)-T cells, (CAR)-natural killer (NK) cells and tumor-infiltrating lymphocytes. Furthermore, immune checkpoint inhibitors, bi-specific T-cell engagers and cancer vaccination strategies are under (clinical) evaluation (6). Of these, adoptive cell transfer, and more particularly CAR-T cell therapy holds great promise due to the unseen curative outcomes observed in other hematological malignancies, namely high-grade lymphomas and leukemias (7).

CAR-T cells are patients’ own T-cells that are genetically modified to express a transmembrane CAR (8). This receptor can recognize a tumor antigen, expressed on the surface of tumor cells, with its extracellular domain. Classically, a monoclonal antibody (mAb)-derived single-chain variable fragment (scFv) is incorporated to achieve tumor antigen recognition (9, 10). Upon antigen encounter, the intracellular T-cell co-stimulatory (most often CD28- and/or 4-1BB-derived) and CD3ζ T-cell activation domains are responsible for engaging a cytotoxic T-cell response toward the malignant cell. After *ex vivo* modification and expansion of these patient-derived T-cells, they are administered back to the patient, where they thus act as a living drug (11).

Historically, most of the evolution of CAR design has been focused on the optimization of the intracellular portion of the receptor, in order to achieve maximal T-cell activation (10). The extracellular antigen-binding moiety has received less attention, as many mAbs against relevant tumor antigens are (clinically) available. However, the use of the artificial mAb-derived scFv format has been linked to some limitations in CAR-T cell efficacy (9, 12). Firstly, scFvs lack natural stability and therefore need to be artificially linked. This instability has been associated with aggregation, antigen-independent (tonic) signaling and subsequent premature T-cell exhaustion (13, 14). Secondly, their non-human nature limits CAR-T cell persistence *in vivo*, as anti-CAR immune responses have been observed in patients upon relapse (15). Thirdly, because these molecules are usually derived from clinically validated mAbs, there is often no step of (structural) optimization of the scFv domain included in the development of these CARs, as a scFv selection procedure is not required (9, 11). In recent years however, the importance of optimizing this extracellular protein domain has become increasingly recognized, and alternatives to the classical scFv format are rapidly emerging, as reviewed elsewhere (9). One example of such alternative CAR design incorporates a Variable Heavy domain of Heavy chain (VHH) molecule, derived from camelid-found heavy-chain-only antibodies (HCAbs) as an antigen-binding moiety. HCAbs compare to mAbs by lacking light chains and constant heavy-1 (C_H_1) domains (16). The antigen-binding part therefore consists of only one protein domain, the VHH. Evolutionarily, this has ensured that the VHH domain behaves as a monomer and is therefore intrinsically more stable compared to a scFv (16). It also allows for more straightforward VHH screening and selection, as the availability of (immune) libraries is more evident (16). Furthermore, VHH immunogenicity is expected to be lower in comparison to scFvs (17), as there is a high sequence resemblance with human VH sequences of family III. At this point, no reports of anti-VHH CAR immune responses with a neutralizing effect on CAR-T cell therapy have been reported from clinical trials (18), and VHH humanization protocols are available (9, 19, 20).

In the last decades, CD19-directed CAR-T cell therapy has astonished the medical landscape with its curative outcomes in certain forms of leukemia and lymphoma (21). This raises hope for its potential in other hematological malignancies, including MM (11). However, as CD19 is usually not expressed by plasma cells, clinical success of these well-established CAR-T cell products in MM has been limited (11). Higher success rates have been achieved with B-cell maturation antigen (BCMA)-targeted CAR-T cells, with currently two FDA-approved CAR-T cell products (i.e., idecabtagene vicleucel and ciltacabtagene autoleucel) as a result (7). Although the observed overall response rates are high, a progression-free survival period of more than 1 year is observed in under 50% of patients treated (22, 23). The most commonly reported causes for this are antigen shedding and CAR immunogenicity, which can lead to anti-CAR immune responses with a therapy-neutralizing effect (11, 18, 22). Hence, other target proteins are under investigation. Of these, SLAMF7/CS1, GPRC5D, CD138 and CD38 are showing different clinical success rates, while data for newer tumor antigens like CD70, NKG2DL and κ-light chain are to be expected (11). Particularly of interest as an alternative cancer antigen for MM CAR-T cell therapy is CD2 subset 1 (CS1, CD319 or signaling lymphocytic activation molecule family member 7 [SLAMF7]). Indeed, CS1 is highly expressed by > 95% of both healthy as well as malignant plasma cells (11, 24), and expression is retained after multiple lines of therapy (25). Expression on healthy tissue is lower, limited to hematopoietic cell lineages (NK, T, B, and dendritic cells; monocytes and macrophages) and absent on hematopoietic stem cells (11, 26).

These observations have provided the rationale for this study, which consists of developing a new form of CAR-T cell therapy for MM, targeting CS1. To this end, we aimed to develop an optimized CAR through variation in the antigen-binding part of the receptor, as well as determine which specific parameters of this extracellular part are crucial to achieve potent CAR-T cell activation. The influence of differences in affinity, cell-binding capacity, epitope location, cell-to-cell distance and binding orientation were examined as possible determinant factors.

## 2 Materials and methods

### 2.1 Cell culture

Bacterial cell lines used include *E. coli* TG1 (Sigma Aldrich), *E. coli* WK6 (American Type Culture Collection, ATCC), *E. coli* NEB5-α (New England BioLabs) and *E. coli* XL1-Blue (Agilent) and were all cultured in lysogeny broth. Mammalian cell lines used include cancer cell lines OPM2 (CS1^pos^, ATCC), JJN3 (CS1^neg^, ATCC) and murine CS1^pos^ 5T33vt, described before (27) –, which were all cultured in Roswell Park Memorial Institute 1640 culture medium (Gibco), supplemented with 10% (v/v) fetal bovine serum, 2 mM L-Glutamine, 1% (v/v) penicillin-Streptomycin, 1% (v/v) non-essential amino acids and 1 mM sodium pyruvate, all from Thermo Scientific. The lentiviral vector (LV) production cell line HEK293T was obtained from ATCC and cultured in Dulbecco’s Modified Eagle Medium culture medium (Gibco), equally supplemented. Human reporter T-cell line Jurkat-67 (2D3) contains the gene for eGFP under an NFAT-driven promoter, as described before (28), and was cultivated in equally supplemented Iscove’s Modified Dulbecco’s Medium (Gibco).

### 2.2 Identification of CS1-specific VHHs

#### 2.2.1 Immunization and VHH library construction

Recombinant CS1 proteins (CS1-(his)C) were produced and purified by U Protein Express (Utrecht, The Netherlands). Briefly, HEK293-E 253 cells were transiently transfected with DNA encoding the extracellular portion of either human or murine CS1, C-terminally fused to a hexahistidine tag. Purification from the supernatant was ensured by subsequent Immobilized Metal Affinity Chromatography (IMAC) and Size Exclusion Chromatography (SEC). A llama was immunized by weekly injections of a mixture of 100 µg of recombinant human and murine CS1 proteins, combined with Gerbu adjuvant, over a period of 6 weeks. Total mRNA extraction from 10^7^ peripheral blood lymphocytes, isolated from 100 ml blood sample, yielded 40 µg of mRNA which was used to generate the immune VHH phagemid library via procedures described elsewhere (29).

#### 2.2.2 Biopanning

For VHH selection, the VHH library was cloned into a pMECS phagemid vector, as previously described (29). Phages expressing the VHHs on their surface were produced after transformation of *E. coli* TG1 cells and infection with M13 VCS helper phages. Four rounds of biopanning were performed on the biotinylated variant of human CS1 (CS1-(his)C-PEG4-biotin), custom-produced by U Protein Express. As it was intended to select human CS1-specific VHHs, we will further refer to human CS1 as CS1. In each round, these phages were incubated with CS1-(his)C-PEG4-biotin (100 nM in rounds 1 and 2; 10 nM in rounds 3 and 4) and phage selection was performed via magnetic streptavidin beads (New England Biolabs). Phage elution was obtained with 50 mM dithiothreitol. Harvested phages were infected into *E. coli* TG1 cells for VHH production (single colonies) and generation of the VHH sub-library for further rounds of panning.

#### 2.2.3 VHH screening via ELISA

A randomized selection of single *E. coli* TG1 colonies carrying a VHH-pMECS plasmid was made and bacterial colonies were produced at 2 ml culture scale in lysogeny broth medium. Periplasmic production of hexahistidine- and hemagglutinin (HA)-tagged VHHs was induced with 1 mM isopropyl β-D-1-thiogalactopyranoside (IPTG). Crude periplasmic extracts were obtained via freeze-thawing and CS1 binding was evaluated in an enzyme-linked immunosorbent assay (ELISA) on 100 ng recombinant CS1, coated on Nunc MaxiSorp plates (Invitrogen). This by subsequent staining with murine anti-HA-tag mAb (Sigma Aldrich) and alkaline phosphatase-coupled goat anti-mouse mAb (Sigma Aldrich), as described (29, 30). A tripling of the 405 nm absorption signal compared to background was used as a threshold to identify candidates as positive. Positive VHH clones were sequenced.

### 2.3 Selection and production of CS1-specific VHHs

#### 2.3.1 Flow cytometry

VHH binding to cell-expressed CS1 protein was evaluated in flow cytometry on CS1^pos^ MM cells (OPM2). Periplasmic extract was used, containing hexahistidine- and HA-tagged VHHs at unknown concentration, of which cell binding was detected by subsequent staining with mouse-anti-HA IgG1 mAb and phycoerythrin (PE)-labeled anti-mouse IgG1 mAb (BD Biosciences). Cell fluorescence was measured on a FACSCanto Flow Cytometer (BD Biosciences). Data analysis was performed using the FlowJo 10.9.0 software (BD Biosciences).

#### 2.3.2 Off-rate screening

The off-rate dissociation constant of binding (k_d_) is concentration-independent and can therefore be determined using surface plasmon resonance (SPR) technology on periplasmic extract. To this end, biotinylated recombinant CS1 protein (U Protein Express) was coated on a streptavidin chip in a Biacore T200 instrument. Periplasmic extract in hepes-buffered saline (HBS) was run over the chip and dissociation was monitored for 600 s in HBS. The chip was regenerated using 0.1 M glycine HCl (pH 2.0) between different measurements. Evaluation was performed using the Biacore T200 2.0 evaluation Software (GE Healthcare).

#### 2.3.3 VHH production and purification

VHHs were cloned in the pHEN6c production vector (31) via described procedures (29). These pHen6c vectors were transformed into chemo-competent *E. coli* WK6 cells and periplasmic VHH expression was induced using 1 mM IPTG. Periplasmic extracts were obtained via osmotic shock and from there hexahistidine-tagged VHHs were purified by subsequent IMAC and SEC – as described (29). A non-targeting control VHH (R3B23)- described elsewhere (32)-, was produced in parallel, following identical procedures.

### 2.4 Molecular and *in vivo* characterization of purified CS1-specific VHHs

#### 2.4.1 Flow cytometry

To detect VHH binding to cell-expressed antigen, CS1^pos^ OPM2 cells were incubated with the VHHs at 200 nM for 1 h at 4°C. Detection was performed by staining for the C-terminal hexahistidine tag using a primary mouse anti-His IgG1 mAb (Biolegend), followed by a secondary allophycocyanin (APC)-labeled anti-mouse IgG1 mAb (Biolegend). A positive control for antigen-expression by the target cells (APC-mouse anti-human CS1 IgG2bκ mAb; Biolegend) and its isotype control (APC-mouse IgG2bκ isotype control; Biolegend) were included. All staining steps were performed according to manufacturer’s instructions. Cell-fluorescence was evaluated using the BD FACSCelesta Cell Analyzer (BD Biosciences) and data analysis was done with the FlowJo 10.9.0 software (BD Biosciences).

#### 2.4.2 Affinity determination via SPR

Kinetic parameters for VHH binding to CS1 were determined via SPR on a Biacore T200 instrument (GE Healthcare). To that end, 5 μg/ml recombinant CS1 protein in 10 mM sodium acetate, pH 4.0 (VWR International) was coupled on a 100 mM N-hydroxysuccinimide (GE Healthcare) and 400 nM 1-ethyl-3-(3-dimethylaminopropyl) carbodiimide hypochloride (GE Healthcare)-activated CM5 sensor chip. Blocking of residual binding sites was achieved with 1 M ethanolamine-HCl (GE Healthcare). A 1/2 VHH dilution series of the purified VHHs, ranging from 100 nM to 3.125 nM (with duplicate at 50 nM), in HBS (pH 7.4) was run over the CS1-coated chip at 25°C. Binding association was allowed for 180 s and dissociation for 300 s. CM5 chip regeneration was ensured by 0.1 M glycine HCl (Sigma Aldrich) at pH 2.0. The association (k_a_), dissociation (k_d_) and equilibrium dissociation constants (K_D_) were calculated with the Biacore T200 2.0 evaluation Software (GE Healthcare) using the ‘1:1 binding with drift and RI2’ fitting model.

#### 2.4.3 ^99m^Tc radioactive labeling, *in vivo* microSPECT/CT imaging and *ex vivo* biodistribution

After sedation via isoflurane inhalation (Vetflurane, Virbac; 5% induction and 2.5% maintenance), 10x10^6^ CS1^pos^ OPM2 cells in 100 µl phosphate buffered saline (PBS) were inoculated subcutaneously in the right flank of female, 6-week-old CB17/lcr-*Prkdc^SCID^*/lcrlcoCrl mice (Charles River). At 18-19 days post inoculation, tumors were palpable (100 - max. 500 mm^3^), allowing biodistribution studies. To this end, hexahistidine-tagged VHHs were site-specifically radiolabeled with ^99m^Tc, as previously described (33). Next, 100 µl of the ^99m^Tc -labeled VHHs was injected intravenously in isoflurane-sedated animals. At 50 min post injection (p.i.), mice were sedated with 75 mg/kg ketamine (Ecuphar) + 1 mg/kg medetomidine (Virbac) via intraperitoneal injection and subjected to a 2 min microCT and a 20-minute pinhole-SPECT scan at 1 h p.i. of the radiotracer. Image analysis was performed with the AMIDE 1.0.4. and OsiriX MD 11.0 software. Sedated animals were sacrificed via neck dislocation after imaging. Organ collection for *ex vivo* biodistribution analysis was performed at 90 min p.i. and radioactive uptake in selected organs was measured using a γ-counting instrument (2480 WIZARD2 Automatic Gamma Counter, Perkin Elmer). Data were normalized to organ weight and corrected for radioactive decay. All animal experiments were approved by the ethical committee for use of laboratory animals of the Vrije Universiteit Brussel (Brussels, Belgium) (license number 19-281-2).

### 2.5 Generation of VHH-CAR encoding lentiviral particles (LVs)

#### 2.5.1 Cloning

**Figure 1:**
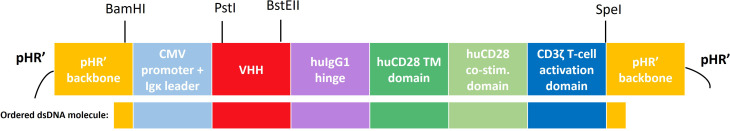
Schematic overview of the CAR construct and its subdomains the pHR’-derived lentiviral transfer plasmid. A second-generation human CAR was used, incorporating an Igκ leader sequence, a cytomegalovirus (CMV) promoter, a CS1-specific or non-targeting control VHH, a hinge region derived from human IgG4, a transmembrane and intracellular co-stimulatory domain derived from human CD28 and a human CD3ζ-derived T-cell activation domain. Relevant (unique) restriction sites are indicated above. Below the construct, the part that was ordered as a dsDNA molecule (gBlock) from Integrated DNA Technologies (IDT) is highlighted. CMV, cytomegalovirus; TM, transmembrane; hu, human; co-stim., co-stimulatory.

To generate a CAR-encoding pHR’-derived lentiviral transfer plasmid, a dsDNA oligonucleotide molecule containing a second-generation CAR sequence with adequate pHR’ vector overhangs of 20 nucleotides was designed and ordered to-demand from Integrated DNA Technologies (IDT). This gBlock was assembled into a BamHI/SpeI-linearized (Thermo Scientific) pHR’-derived lentiviral transfer plasmid (described before by Breckpot et. al. (34)), via the Gibson Assembly Method (IDT). This ensured integration of the CAR sequence in the triple helix and 3’ ΔU3 long terminal repeat-containing pHR’ backbone, upstream of an Internal Ribosomal Entry Site (IRES) and a truncated Nerve Growth factor Receptor (tNGFR) reporter gene (as described (34)) and downstream of a cytomegalovirus (CMV) promoter. The lay-out of the CAR backbone is depicted in **Figure 1**.

VHH cloning into the lentiviral CAR backbone was performed via PstI/BstEII restriction from the pHEN6c production vectors, followed by T4 DNA ligation (Thermo Scientific), according to manufacturer’s instructions. Resulting transfer plasmids were transformed into commercially available competent NEB5-α *E. coli* (New England Biolabs) and subsequently into XL1-Blue *E. coli* (Thermo Scientific) for larger scale plasmid production. Transformation was executed using the TransformAid Bacterial Transformation Kit (Thermo Scientific). Large scale plasmid purification was performed using the NucleoBond Xtra Maxi kit (Macherey-Nagel). Quality control included plasmid yield and purity assessment via OD_260_/OD_280_ measurements on a Implen NanoPhotometer (Westburg), gel electrophoresis to verify plasmid integrity and correct restriction digestion (1.5% agarose gel, Mupid One Electrophoresis apparatus, Advance Co. Ltd.), and sequence verification (NightXpress Mix2Seq Kit, Eurofins Genomics).

#### 2.5.2 HEK293T cell transfection

Transfer plasmids were co-transfected with the envelope-encoding plasmid pMD.G and the packaging plasmid pCMVΔR8.9 at a 3:1:2-ratio into HEK293T cells, as described before (34, 35). Both the pMD.G and pCMVΔR8.9 plasmid were a kind gift of D. Trono (University of Geneva Medical School, Geneva, Switzerland). Culture supernatant containing lentiviral particles (LVs) was harvested at 48 h and 72 h after transfection.

#### 2.5.3 LV concentration

Subsequent 0.22 µm filtration and ultracentrifugation for 90 min at 64074 x g (Beckman SW28 rotor; Optima LE-80K ultracentrifuge; Beckman Coulter) of the culture supernatant was performed to pellet and concentrate the LVs, which were resuspended in PBS supplemented with 10 µg/ml protamine sulphate (LeoPharma). LV titers were determined by titration of a serial dilution on HEK293T cells, as described (36). Evaluation of transduction was done at 72 h post transduction via flow cytometry using the BD FACSCelesta Cell Analyzer, after staining for the tNGFR reporter protein with APC-coupled anti-NGFR mAb; Biolegend). Data analysis was done with the FlowJo 10.9.0 software (BD Biosciences) and titers were calculated using following formula:


$$\frac{\#cells\;at\;time\;of\;transduction\;x\;fraction\;of\;infected\;cells\;x\;dilution\;factor}{transduction\;volume\;in\;ml}$$

### 2.6 2D3 reporter T-cell transduction and selection of stable 2D3-CAR cell lines

Transduction was obtained by incubating 10^5^ 2D3 reporter T-cells with the LVs at a multiplicity of infection (MOI) of 10 in culture medium enriched with 10 μg/ml protamine sulphate (28). Transduction efficiency was evaluated in flow cytometry via incubation with biotinylated CS1 antigen (U Protein Express), followed by PE-coupled streptavidin (eBioscience). Cell fluorescence was measured on a BD FACSCelesta instrument and data analysis was done with the FlowJo 10.9.0 software (BD Biosciences). Cell lines with a stable transduction rate of > 95% were obtained by fluorescence activated cell sorting (FACS), after staining with biotinylated CS1 antigen and PE-coupled streptavidin. FACS was performed on a BD FACSAria™ III Cell Sorter. 2D3 cells stably expressing a VHH-CAR on the surface are further on denoted as 2D3-[VHH number].

### 2.7 Evaluation of T-cell activation potential by different VHH-CARs

#### 2.7.1 CAR-T cell activation assay

2D3-CAR cells were co-cultured with OPM2 (CS1^pos^) or JJN3 (CS1^neg^) target MM cells (referred to as the stimulated and unstimulated condition, respectively) at an E:T ratio of 10:1 in supplemented IMDM culture medium at 37°C, 5% CO_2_. Green fluorescence was followed-up in real-time in the IncuCyte ZOOM apparatus over a period of 40 h. Subsequently, cells were stained with biotinylated CS1 protein (U Protein Express) for CS1-specific 2D3-CARs or biotinylated anti-VHH mAb (GenScript) for 2D3-R3B23, followed by PE-coupled streptavidin for further analysis in flow cytometry. Antigen expression by the target cell lines was confirmed in flow cytometry after staining with APC-labeled anti-CS1 IgG2bκ mAb (Biolegend) and APC-labeled IgG2bκ isotype control mAb (Biolegend). Flow cytometry measurements were performed on a BD FACSCelesta apparatus (BD biosciences) and data analysis was done with the FlowJo 10.9.0 software (BD Biosciences).

#### 2.7.2 Co-culture competition assays

MM cells were pre-incubated with a 1 µM saturating concentration of different soluble VHHs (1 h, 37°C, 5% CO_2_), after which 2D3-CAR cells were added at a 1:1 ratio. Evaluation of CAR T-cell activation was performed via flow cytometry as described above at 40 h post co-incubation.

### 2.8 Estimation of binding orientation by *in silico* modeling

*In silico* 3D simulations of VHH-CAR:CS1 interactions were made via the online AlphaFold2 (v1.5.2) software (37). Evaluation of obtained structure predictions was performed in PyMol (v.4.6.0). To estimate the binding distance, distances were determined between 1) the C-terminal serine residue S(128) of the VHH and the most membrane-distal point of CS1 D(49); and 2) the C-terminal serine residue S(128) of the VHH and CS1 membrane anchor point A(219). Using the Pythagorean theorem, the distance between the CAR anchor point of the VHH S(128) and the membrane anchor point of CS1, as projected onto the axis of the extracellular part of CS1, was calculated. Bindings angles were calculated between the most membrane-distal residue of CS1 D(49); the membrane anchor point of CS1 A(219) and the terminal serine in the VHH S(128). A visual representation of the determined distances and angles is provided in **Figure 6C**.

### 2.9 Statistical analysis

Statistical analysis of *ex vivo* biodistribution experiments was performed by one-way ANOVA (multiple t-tests) in which each CS1-specific VHH was compared to the non-targeting control VHH R3B23, described before (32). For the analysis of the CAR activation assay, the difference between the stimulated and unstimulated condition was used as a measure of (specific) T-cell activation. Via one-way ANOVA (multiple t-tests), each antigen-specific VHH-CAR was compared to the non-targeting control VHH-CAR R3B23. Additional information about the number of replicates for each assay is provided in the adequate figure legends. All analyses were performed using the GraphPad Prism 9.1.0 software. *, P<0.05; **, P<0.01; ***, P<0.001; ****, P<0.0001; not significant (n.s.), P>0.05.

## 3 Results

### 3.1 Identification of anti-CS1 VHHs and evaluation of affinity characteristics

For CS1-specific VHH identification, a VHH immune phage display library was constructed after llama immunization with recombinant human and murine CS1 proteins. This library was displayed on the tip of bacteriophages and then subjected to four rounds of biopanning in solution on biotinylated CS1 protein.

**Figure 2:**
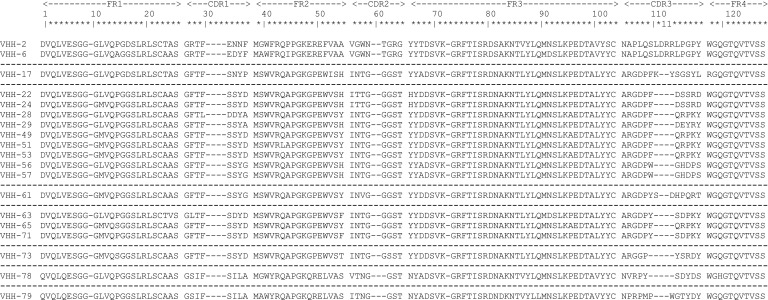
Overview of the protein sequences of the 19 unique CS1-specific VHHs identified. Residue numbering is displayed according to the standard ImMunoGeneTics (IMGT) numbering system (38). Different VHH families are separated by dashed lines. FR, framework region; CDR, complementarity-determining region.

After panning, a randomized selection of 285 VHH clones from different rounds of selection was produced in crude form, which was screened in ELISA for binding to immobilized recombinant CS1 protein. From these, 254 were considered positive for binding. Sequencing revealed these to be 81 unique VHH molecules. Flow cytometry analysis showed that all identified subjects bind CS1^pos^ OPM2 cells, and a subsequent off-rate screening confirmed these observations. From these 81 identified clones, 19 VHHs, belonging to eight different VHH families - according to the standard ImMunoGeneTics (IMGT) numbering system (38) -, were selected for production, purification, and in-depth characterization (**Figure 2**). The parameters used for this selection were the observed off-rate (k_d_), sequence differences, the presence of stop codons in the sequence, as well as sequence prevalence.

**Figure 3:**
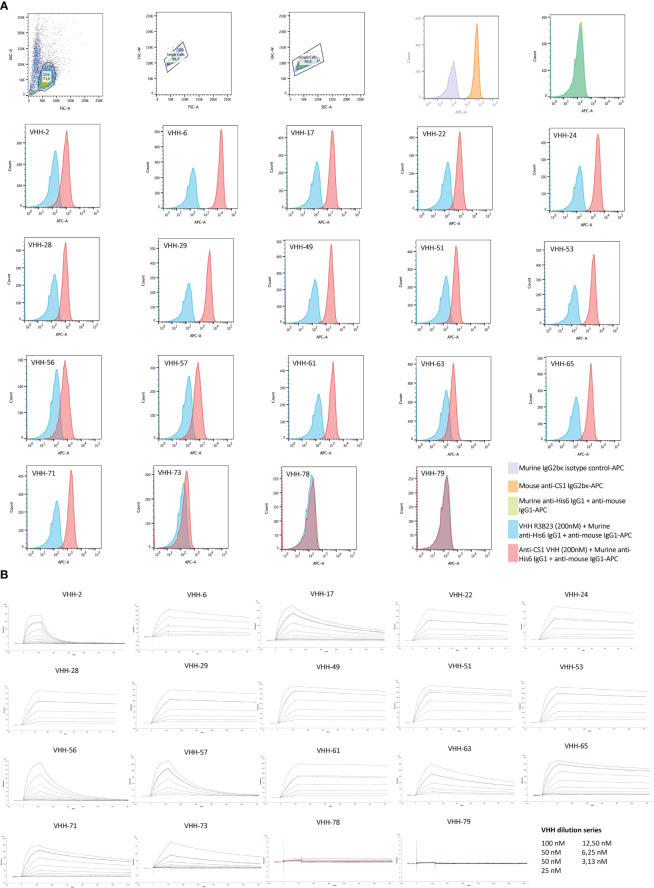
Binding characteristics of the selected CS1-specific VHHs. **(A)**: Flow cytometry results to confirm VHH binding to cell-expressed CS1 protein on CS1^pos^ OPM2 MM cells. Cell binding of the CS1-specific VHHs at 200 nM is shown by the red histograms, relative to binding of the non-targeting control VHH R3B23 at 200 nM (blue histograms). CS1 expression by the target OPM2 cells was confirmed with an APC-labeled anti-CS1 mAb (orange histogram), relative to its APC-labeled isotype control mAb (purple histogram). The situation in which no VHH was added to the cells is displayed by the green histogram (n=1); **(B)**: Sensograms showing VHH association and dissociation from a CS1-coated CM5 sensor chip in SPR at different VHH concentrations, in a 1/2 dilution series ranging from 100 nM to 3.13 nM with a duplicate measurement at 50 nM. From this, kinetic binding parameters k_a_, k_d_ and K_D_ were calculated, using the ‘1:1 binding with drift and RI2’ fitting model in the Biacore T200 2.0 evaluation Software (GE Healthcare), (n=1).

Further characterization of the produced and purified VHHs involved screening in flow cytometry and affinity determination via SPR. Out of the 19 candidates, 17 VHHs were confirmed to bind cell-expressed CS1 in flow cytometry to different extents (**Figure 3**; **Table 1**). These findings were confirmed in SPR experiments (**Figure 3B**), where VHH-2 to VHH-73 showed binding to human CS1 with an affinity range of K_D_ = 0.33 nM (VHH-61) to K_D_ = 49.70 µM (VHH-2), as summarized in **Table 1**. The two non-binding compounds VHH-78 and VHH-79 were identified as murine CS1 (muCS1) binding VHHs in follow-up SPR experiments on plate-coated muCS1 (**Supplementary Figure 1**) and flow cytometry experiments on muCS1^pos^ 5T33vt cells (**Supplementary Figure 2**). None of the identified VHHs were cross-reactive for human and murine CS1.

**Table 1:** Summary of the affinity parameters of the produced CS1-specific VHHs.

VHH	ΔMFI [VHH] = 200 nM	K_D_ (nM)	k_a_ (M^-1^s^-1^)	k_d_ (s^-1^)
**2**	**6.09**	**49.72**	**6.41 x 10^5^**	**3.18 x 10^-2^**
**6**	**101.30**	**3.80**	**2.10 x 10^5^**	**7.90 x 10^-4^**
**17**	**13.03**	**9.01**	**3.66 x 10^5^**	**2.99 x 10^-1^**
22	7.29	4.00	1.20 x 10^5^	4.80 x 10^-4^
24	20.61	4.70	1.00 x 10^5^	4.70 x 10^-4^
28	6.58	1.20	2.10 x 10^5^	2.50 x 10^-4^
**29**	**29.45**	**1.60**	**1.50 x 10^5^**	**2.40 x 10^-4^**
51	14.29	0.60	4.90 x 10^5^	2.90 x 10^-4^
56	5.33	0.94	3.00 x 10^5^	2.80 x 10^-4^
**53**	**22.09**	**0.81**	**3.70 x 10^5^**	**3.00 x 10^-4^**
56	4.72	36.07	2.79 x 10^5^	1.00 x 10^-2^
**57**	**4.80**	**24.70**	**3.50 x 10^5^**	**8.50 x 10^-3^**
**61**	**10.34**	**0.33**	**2.50 x 10^5^**	**8.10 x 10^-5^**
**63**	**3.37**	**9.80**	**1.90 x 10^5^**	**1.90 x 10^-3^**
65	11.43	2.32	4.23 x 10^5^	9.79 x 10^-4^
**71**	**11.24**	**4.29**	**3.72 x 10^5^**	**1.60 x 10^-3^**
**73**	**1.35**	**35.76**	**7.44 x 10^4^**	**2.54 x 10^-3^**
78	2.07	*N.A.*	*N.A.*	*N.A.*
79	0.77	*N.A.*	*N.A.*	*N.A.*

Shift in Mean Fluorescence Intensity (ΔMFI) is determined as the difference in MFI measured in flow cytometry between the CS1-specific VHH at a concentration of 200 nM and the nontargeting control VHH R3B23 at an equal concentration on CS1pos OPM2 cells (n=1). Kinetic binding parameters of VHHs toward plate-coated antigen (k_a_, k_d_, and K_D_) are determined via SPR on recombinant CS1 protein (n=1). VHHs that were selected for further evaluation are indicated in bold. N.A. = not applicable; SPR, surface plasmon resonance.

Taken these results, as well as sequence differences and VHH production yields into consideration, a further selection of 10 VHHs was done, i.e*.*, VHHs 2, 6, 17, 29, 53, 57, 61, 63, 71 and 73 (highlighted in bold in **Table 1**).

### 3.2 In line with their affinity for CS1, different VHHs target CS1^pos^ MM tumors to a different extent *in vivo*

**Figure 4:**
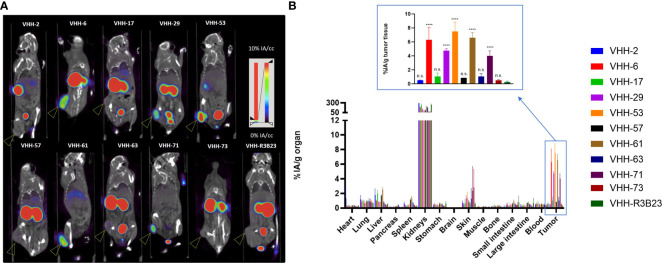
Radiolabeled anti-CS1 VHHs target MM tumors in mice to different extents. **(A)**: Representative microSPECT/CT images of tumor-bearing mice at 1 h p.i. with ^99m^Tc-radiolabeld anti-CS1 VHHs (n = 3). Subcutaneous OPM2 tumors are indicated with an arrow. **(B)**: *Ex vivo* biodistribution data, obtained at 90 min p.i. with ^99m^Tc-radiolabeld CS1-specific VHHs. Graphs show the mean ± standard deviation (SD) radioactive uptake, normalized to organ or tissue weight and corrected for radioactive decay (n = 3). P-values are determined using one-way ANOVA (multiple t-tests) in which each CS1-specific VHH was compared to the non-targeting control VHH-R3B23. *P< 0.05; **P< 0.01; ***P< 0.001; ****P< 0.0001; not significant (n.s.), P > 0.05.

We next investigated whether the *in vitro* affinity parameters of anti-CS1 VHHs also translated into an *in vivo* tumor-targeting capacity. To this end, the VHHs were site-specifically radiolabeled with ^99m^Tc on their C-terminal hexahistidine tag. Radioactive compounds were next injected intravenously into subcutaneous MM-bearing (CS1^pos^ OPM2 cells) mice. A 20 min microSPECT/CT scan was performed at 1 h p.i. to visualize the *in vivo* biodistribution in living animals (**Figure 4A**). At 90 min p.i., animals were sacrificed to quantify radioactive uptake in isolated organs. Tumor uptake ranged from 0.48 ± 0.07% injected activity (IA)/g for VHH-2 to 7.50 ± 1.35% IA/g for VHH-53, with uptake of the non-targeting control VHH-R3B23 being 0.27 ± 0.12% IA/g (**Figure 4B**).

A statistically relevant elevated tumor uptake (p-value < 0.0001) compared to the non-targeting control VHH-R3B23 was observed for VHH-6 (6.29 ± 1.81% IA/g), VHH-29 (4.75 ± 0.29% IA/g), VHH-53 (7.50 ± 1.35% IA/g), VHH-61 (6.57 ± 0.79% IA/g) and VHH-71 (4.02 ± 0.71% IA/g). Intermediate affinity VHH-6, VHH-29 and VHH-71 (5 nM > K_D_ > 1 nM) equally show mediocre tumor accumulation, whereas high affinity VHH-53 and VHH-61 (K_D_ < 1 nM) are the most potent tumor-targeting compounds.

These experiments further revealed that compounds displaying a weaker affinity for the target protein (K_D_ > 5 nM, as measured in SPR, **Table 1**), are the ones showing the lowest tumor uptake *in vivo*. This accounts for VHH-2, with a tumor uptake of 0.48 ± 0.07% IA/g (p-value = 0.9995), VHH-17 (1.05 ± 0.50% IA/g, p-value = 0.8001), VHH-57 (0.85 ± 0.01% IA/g, p-value = 0.9526), VHH-63 (1.03 ± 0.45% IA/g, p-value = 0.8173) and VHH-73 (0.52 ± 0.08% IA/g, p-value = 0.9994). These correlations highlight the importance of a strong affinity of the VHHs toward their target antigen to achieve adequate tumor targeting *in vivo*. Equally was the tumor-to-muscle-uptake elevated for VHH-6, VHH-29, VHH-53, VHH-61 and VHH-71, being the intermediate-to-high affinity compounds. Tumor-to-blood-uptake ratios were elevated for VHH-53, VHH-61 and VHH-71 and the tumor-to-bone ratios for VHH-6 and VHH-53 (**Table 2**). As expected due to the renal clearance route of VHH-sized compounds, accumulation in the kidneys was high.

**Table 2:** Tumor-to-blood, -bone and -muscle radioactive uptake ratios of anti-CS1 VHHs.

VHH	Tumor/blood		Tumor/bone		Tumor/muscle	
**2**	1.08 ± 0.02	ns	2.72 ± 0.68	ns	3.32 ± 0.30	ns
**6**	11.07 ± 3.94	ns	34.83 ± 18.65	*	35.86 ± 19.01	*
**17**	1.79 ± 0.13	ns	2.67 ± 0.77	ns	8.75 ± 4.71	ns
**29**	10.17 ± 1.85	ns	17.31 ± 0.29	ns	73.46 ± 32.23	****
**53**	17.01 ± 6.30	**	45.17 ± 33.39	**	45.63 ± 15.43	**
**57**	1.68 ± 0.30	ns	3.13 ± 1.02	ns	4.52 ± 1.12	ns
**61**	14.23 ± 3.07	*	21.30 ± 8.79	ns	39.26 ± 8.85	*
**63**	2.26 ± 0.36	ns	6.07 ± 3.08	ns	5.49 ± 0.30	ns
**71**	18.21 ± 13.25	**	25.68 ± 8.52	ns	34.10 ± 7.79	*
**73**	1.30 ± 0.27	ns	5.97 ± 2.99	ns	3.01 ± 0.01	ns
**R3B23**	0.44 ± 0.21		1.52 ± 0.94		1.70 ± 0.78	

These values are calculated using the *ex vivo* biodistribution data. P-values are determined using one-way ANOVA (multiple t-tests) in which each CS1-specific VHH was compared to the nontargeting control VHH-R3B23 (n=3). *P< 0.05; **P< 0.01; ***P< 0.001; ****P< 0.0001; not significant (n.s.), P > 0.05.

### 3.3 The CAR-incorporated VHH has an affinity-independent influence on T-cell activation kinetics

**Figure 5:**
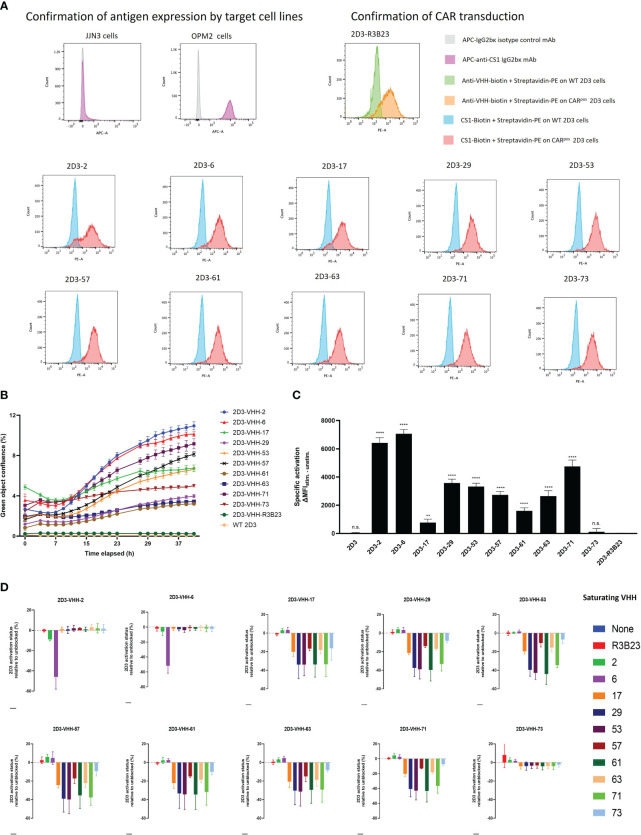
CS1-specific VHH-CAR-T cell activation assessment. **(A)**: Confirmation of antigen expression by the target cell lines (JJN3 and OPM2) and CAR-expression by the 2D3 cell lines transduced with the different CS1-specific VHH-CARs and the non-targeting negative control VHH-R3B23-CAR via flow cytometry (n=1). **(B)**: Real-time follow-up of green fluorescence in the IncuCyte Zoom Live-Cell Analysis System of co-cultures of CS1^pos^ OPM2 MM cells with the different CS1-specific 2D3-VHH cell lines, the negative control 2D3-VHH-R3B23 and the wild type (WT) 2D3 cell line at a (1:10) cell ratio. Data are quantified as mean green object confluence (%) ± SD (n=3). **(C)**: CAR-T-cell activation levels as defined by the difference in MFI (ΔMFI) between the stimulated (40 h (1:10) co-cultured with CS1^pos^ OPM2 cells) and the unstimulated (40 h (1:10) co-cultured with CS1^neg^ JJN3 cells) condition, in the CAR^pos^ cell fraction, for each CS1-specific 2D3-VHH-CAR cell line (n=3). Statistical analysis was performed by one-way ANOVA (multiple t-tests), where each CS1-specific 2D3-CAR cell line was compared to the non-targeting control 2D3-CAR R3B23 cell line. *P<0.05; **P<0.01; ***P<0.001; ****P<0.0001; not significant (n.s.), P>0.05. **(D)**: CAR-T cell activation status (defined by the percentage of eGFPpos cells within the CARpos cell fraction) of 40 h (1:1) 2D3-CAR:OPM2 co-cultured cell lines after pre-incubation of the OPM2 cells with a 1 μM excess of soluble VHH for 1 h at 37°C. Data are quantified in terms of percentage activated cells (i.e., eGFPpos cells within the CARpos cell fraction) ± SD after normalizing to the condition in which no soluble VHH is added to the co-culture (n=3). WT, wild type (untransduced cells); MFI, mean fluorescence intensity.

It was next investigated which of these VHHs ensured good T-cell activation when incorporated in a CAR (**Figure 1**). To that end, 2D3 reporter T-cells were used. These are Jurkat-76 cells that express enhanced green fluorescent protein (eGFP) in response to CD3ζ signaling, as they express eGFP under control of a nuclear factor of activated T-cells (NFAT)-driven promotor. 2D3 cells were stably transduced with the different VHH-CARs (**Figure 5A**). As flow cytometry staining was performed by addition of biotinylated antigen followed by PE-labeled streptavidin, the observed shift in mean fluorescence intensity (MFI) is affinity-dependent. Hence, the relatively lower shift in fluorescence observed for VHH-2, in line with its inferior affinity for CS1 (**Table 1**). This method of staining simultaneously confirms the functional expression of the CAR on the surface of the transduced cells, and the appropriate protein folding of the incorporated VHH. The observed transduction rates were confirmed to be stable over time.

Next, the different 2D3-CAR cell lines were co-cultured with either huCS1^pos^ (OPM2) or huCS1^neg^ (JJN3, **Figure 5A**) MM target cells for a period of 40 h (referred to as the stimulated and unstimulated condition, respectively) and expression of eGFP was monitored in real-time using IncuCyte Zoom Live cell analyses (**Figure 5B**). It appeared that VHH-2 and VHH-6 were the most potent CAR T-cell activators, while VHH-61, VHH-63 and VHH-29 showed the least antigen-specific eGFP upregulation, *i.e.*, CAR*-*T cell activation, even though especially VHH-29 and VHH-61 belong to the highest affinity binders toward CS1 (**Table 1**). This implies that there is no direct link between affinity and CAR-T cell activation potency. As expected, neither untransduced (wild type, WT), nor VHH-CAR-R3B23-transduced 2D3 cells displayed upregulation of eGFP when co-cultured with either of the target cell lines. Also, no upregulation of eGFP was observed in any of the CS1-specific 2D3-CAR cell lines upon co-cultivation with CS1^neg^ JJN3 cells (**Supplementary Figure 3**).

These results were afterwards confirmed in flow cytometry, where the difference in eGFP expression, measured in terms of MFI in the CAR^pos^ cell fraction, was calculated between the stimulated and the unstimulated condition (ΔMFI). Compared to the non-targeting control cell line 2D3-R3B23 (ΔMFI = 3.33 ± 26.10), only the untransduced 2D3 cell line (ΔMFI = 17.33 ± 50.90) and 2D3-73 (ΔMFI = 114.67 ± 237.58) failed to lead to significant T-cell activation (**Figure 5C**). All other VHHs proved capable of ensuring CAR-T cell activation (p-values < 0.0001, except for VHH-17), be it to different extent. VHH-6 (ΔMFI = 7064.33 ± 312.62) and VHH-2 (ΔMFI = 6425.67 ± 362.95) showed the most potent activation, confirming IncuCyte observations. These are followed by VHH-71 (ΔMFI = 4752.00 ± 451.60), VHH-29 (ΔMFI = 3575.33 ± 275.65), VHH-53 (ΔMFI = 3326.33 ± 238.55), VHH-57 (ΔMFI = 2731.67 ± 246.73) and VHH-63 (ΔMFI = 2653.00 ± 384.05) that enabled mediocre CAR-T cell activation. VHH-61 (ΔMFI = 1606.00 ± 211.17) and VHH-17 (ΔMFI = 777.00 ± 235.11, p-value = 0.0096) were the least capable of initiating antigen-specific CAR-T cell activation. No direct link between the capability of initiating CAR-T cell activation and any of the specific VHH parameters described above (affinity, cell-binding capacity or *in vivo* tumor-tracing capability) was observed.

### 3.4 Competition studies suggest a role for the VHH-bound epitope in CAR-T activation, which cannot be confirmed by *in silico* VHH-antigen interaction modeling

Since no direct link was observed between the capacity of 2D3 cell activation by the different VHH-CARs and the affinity, the cell-binding capacity or the *in vivo* behavior of the VHHs, the influence of the VHH-bound epitope was next investigated. To this end, competition co-culture assays were set up in which an excess of a particular soluble VHH was added to the 2D3-VHH-CAR:target cell co-culture. In case the addition of soluble VHH causes a reduction or blockage of another VHH-based CAR-T cell’s activation (i.e., competition measured by a lowering of eGFP signal), this reflects their binding capacity for the same epitope.

It was observed that two groups of VHHs could be distinguished, since competition was observed specifically between VHH-2 and VHH-6 in group 1 and between VHHs 17, 29, 53, 57, 61, 63, 71 and 73 in group 2 (**Figure 5D**). In line, the VHHs belonging to group 1 ensured the highest CAR-T cell activation in the CAR activation assay (**Figures 5B, C**), while the VHHs in group 2 consistently evoked lower activation. These similarities between the VHHs within each group suggest a potential link between the VHH-bound epitope and the capability of CAR-T cell activation.

**Figure 6:**
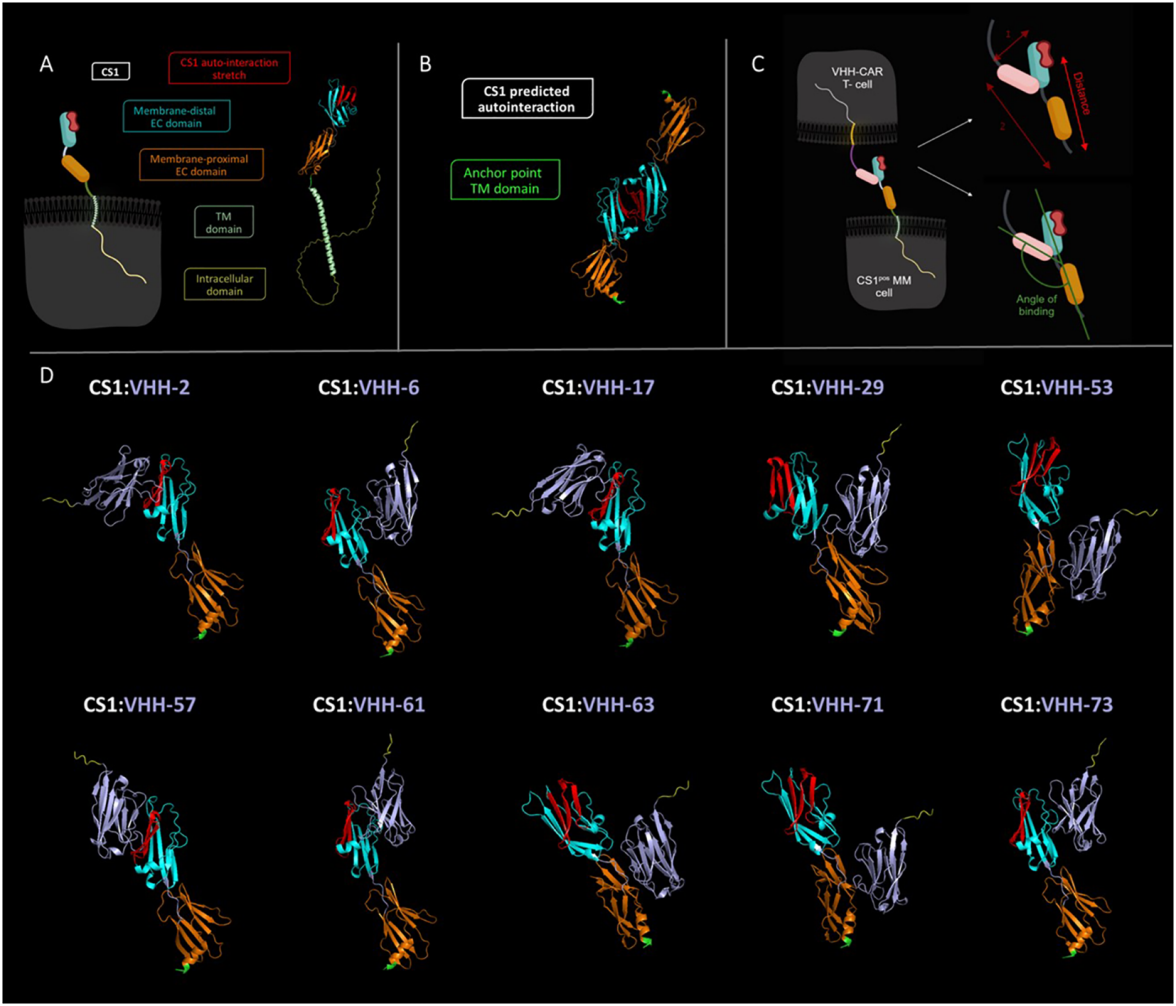
*In silico* predicted structural interaction of the different VHH-CS1 interaction pairs. **(A)**: Left: schematic representation of the cell-expressed CS1 molecule. Right: structural modeling of the CS1 protein. The intracellular part of CS1 is colored yellow, the transmembrane helix is colored in light green, the membrane proximal extracellular domain is shown in orange and the membrane-distal extracellular domain is marked in cyan. The latter contains the predicted protein stretch where CS1 auto-interacts, indicated in red. **(B)**: Structural modeling of the CS1:CS1 auto-interaction. The point where CS1 is membrane-anchored is indicated in green. The membrane-proximal and membrane-distal extracellular domains are displayed in orange and cyan blue, respectively. The auto-interaction protein stretch is colored red. **(C)**: Left: schematic representation of the CS1^pos^ MM cell:VHH-CAR-T cell interaction. Right: Schematic representation of de manner of intercellular distance calculation (top) and of binding angle determination (bottom). The VHH molecule is indicated in pink. **(D)**: Structural modeling of the different VHH-CS1 interaction pairs. VHHs are colored purple with a yellow C-terminal hexahistidine tag. The CS1 membrane-anchoring residue is indicated in green. Modeling was done using the online AlphaFold2 Artificial Intelligence Deep Learning software and images were processed in PyMOL 4.6.0. TM, transmembrane; EC, extracellular. Schematic images shown in panels A and C were created in Biorender.

To verify the hypothesis of the importance of the targeted epitope for CAR-T cell activation, we performed *in silico* modeling of the different VHH-CS1 interactions. For this purpose, the online artificial intelligence-driven self-learning algorithm AlphaFold2 was used, which is able to propose structural protein interactions with a high probability (37, 39), based on sequence information (**Figure 6**). According to this modeling, CS1 consists of an unstructured intracellular domain, a transmembrane alpha helix and two extracellular domains, of which the membrane-distal unit is responsible for the CS1 autointeraction (**Figures 6A, B**). The different hexahistidine-tagged VHHs were modeled together with CS1, from which structural information about the binding interaction was retrieved (**Figure 6D**).

It was assessed whether VHH binding occurred on the membrane-proximal or membrane-distal extracellular domain of CS1 and whether this binding interfered with the CS1 auto-association domain. Furthermore, the distance between the membrane-anchor point of CS1 and the C-terminus of the VHH was calculated, as well as the angle of binding between the VHH-axis and the membrane anchor of CS1 (**Figure 6C**). These may be relevant activation parameters, as they have a direct influence on the intercellular distance between the CAR-T and the target cell. In line with the kinetic segregation model for classical T-cell activation, it has been hypothesized that this is relevant for adequate activation of a CAR-T cell.

**Table 3** summarizes the observations made for the different VHHs. These are listed in order of decreasing 2D3 activation potential (based on the activation values calculated in **Figure 5C**). Four out of the ten VHHs are predicted to bind the membrane-proximal extracellular domain of CS1, while six bind the auto-interaction domain. From these, three compounds also seem to bind a protein stretch overlapping with the CS1 auto-interaction epitope. Binding distances range from 20.31 Å (VHH-71) to 90.16 Å (VHH-57) and the binding angle varies between 76.3° (VHH-17) and 132.2° (VHH-71). No clear link between either of these parameters and the potential of CAR-T cell activation was observed, nor with the data from the competition assay. While the AlphaFold2 algorithm has already proven its predictive power (37, 39), it should be noted here that this information remains prediction-based.

**Table 3:** Summary of the observations made by *in silico* modeling of the different CS1-VHH interaction pairs.

VHH	CS1 binding domain	Overlap with CS1 auto-interaction domain	Binding distance (Å)	Angle of binding (°)	VHH	CS1 binding domain	Overlap with CS1 auto-interaction domain	Binding distance (Å)	Angle of binding (°)
6	Membrane distal	No	82.58	99.0	57	Membrane distal	Yes	90.16	124.0
2	Membrane distal	Yes	68.79	80.1	63	Membrane proximal	No	26.29	129.5
71	Membrane proximal	No	20.31	132.2	61	Membrane distal	No	89.37	120.2
29	Membrane proximal	No	60.25	109.3	17	Membrane distal	Yes	66.38	76.3
53	Membrane proximal	No	41.63	126.6	73	Membrane distal	No	78.18	95.2

For each VHH, it was estimated whether the membrane proximal or –distal extracellular protein domain of CS1 is bound and whether the epitope of VHH binding coincides with the CS1 autointeraction protein stretch. Binding distances were determined between the membrane-anchor point of CS1 and the C-terminus of the VHH, as projected onto the extracellular CS1-axis. The angle of binding was calculated between the axis of each VHH and the extracellular CS1-axis, as represented in **Figure 6C**. Results are displayed in order of decreasing CAR-T cell activation potential (as defined by DMFI in the 2D3 activation assay, determined in **Figure 5C**).

## 4 Discussion

In this study, a side-by-side comparison was made of different VHHs, their characteristics and their ability to lead to T-cell activation when incorporated into a CAR. This was evaluated in the context of MM, a highly relevant cancer type for the optimization of CAR-T cell therapy. We opted to target CS1, often mentioned as a promising next-generation MM antigen, and generated a panel of CS1-specific VHHs, which were fully characterized as soluble compounds and as components of VHH-based CARs.

Although the affinity proved to be predictive for the *in vivo* MM tumor-tracing ability of the VHHs to a certain extent, no link was discovered between the affinity and the ability of CAR-T cell activation. Remarkably even, the relatively weak binding VHH-2 proved to be among the most capable CAR-T cell activating VHHs, while – in line with its affinity for CS1 -, MM cell binding and *in vivo* MM tumor tracing capacity are inferior. A possible link between the targeted epitope of the VHH and CAR-T activation was suggested by competition CAR-T cell activation studies with excess soluble VHH added to the co-culture, but this relationship could not be confirmed by *in silico* structure modeling of the VHH-antigen interactions. The latter was used to estimate whether the intercellular distance and/or the orientation of CAR-target binding influence the T-cell activation potential. Here too, no cause- and-effect relationship was discovered. Of note is that these conclusions were drawn based on the read-out of an eGFP-based reporter T-cell line.

In particular, we have worked with 2D3 cells to assess the influence of the antigen-recognizing domain on T-cell activation. This is an immortalized cell line that is genetically engineered to specifically map (differences in) T-cell activation kinetics (40, 41). In order to make a critical comparison of different binding domains while excluding all other possible factors- biological or other -, a universal screening platform was required. 2D3 cells served that purpose, as they allow the variation of one CAR component solely. Yet, they come with the limitation that no direct estimate can be made of the *in vivo* therapeutic effect and/or possible cytotoxicity of the designed CARs; which are highly important parameters when a clinical translation of the CAR-T cells is envisaged. To further develop the VHH-CARs for therapeutic purposes, *in vivo* evaluation of the compounds is primarily required. However, to estimate the therapeutic potential of the designed CARs in a highly immune-complex disease such as MM, this must be investigated in clinically relevant animal models. These should be fully immune competent and representative of human disease in terms of tumor cell localization, behavior, disease progression and immune system involvement. Such models exist for murine MM disease (42, 43), but are currently not available for the human variant.

Indeed, by making use of a reporter cell line, our data can only state that it is of utmost importance to evaluate a (sub)library of different antigen-binding compounds side-by-side, while already in the structure of a CAR. Therefore, this should be regarded as a proof-of-concept comparison study, aimed at conveying a fundamental message about the importance of multiple candidate-screening when designing CAR-T cells.

In summary, our data simultaneously demonstrate the importance of carefully selecting the extracellular part of a CAR and the serendipity to which this design is subject. Multiple classical VHH parameters were investigated, including affinity, cell binding potential, *in vivo* behavior, epitope location, intercellular T-cell to target cell distance and orientation of the CAR-antigen interaction. Even though important differences were observed in the extent to which different VHHs can activate a CAR-T cell, none of the VHH parameters investigated had a predictive value for this. Therefore, the classical workflow of VHH selection does not match the needs for optimal VHH-CAR design.

Within the medical context, VHHs are mainly used as smaller, soluble, antigen-specific alternatives for the classically large mAbs (44). Therefore, it is important that the compound shows highly discriminative binding to target tissue versus non-target tissue. When certain conditions are met, such as sufficient (*in vivo*) specificity and (thermo)stability, this is generally obtained by compounds that display a high affinity (ideally < 10 nM) for the (cell-expressed) target protein (44, 45). The general flow of the VHH selection protocol, as described here, is considered standard, both by our research group (27, 30, 35, 46, 47), as by related research groups (48, 49), and other groups in the world (50), as well as by industry (45). It is optimized for finding highly stable, soluble pharmaceuticals with strong affinities for target proteins of all kinds, and has resulted in moieties binding different types of proteins, both extracellular (27, 35, 47) and intracellular (51). In fact, this selection procedure is optimized for identifying and selecting *in vivo* tracers. However, the workflow also contains a few bias points for selecting such soluble compounds. Firstly, the initial phase of the selection is performed on crude periplasmic extract, which automatically filters for the highly produced and aqua-stable VHHs in ELISA and flow cytometry. Secondly, an off-rate screening creates bias toward high-affinity compounds with a slow off-rate. Thirdly, thermostability assays and production yield measurements, as well as sequence-dependent parameters are often considered when selecting VHHs. These parameters are less relevant when applications such as VHH-based CAR-T cells are intended.

Instead, it is believed that in order to obtain maximal CAR-T cell activation, the formation of a functional immunological synapse between the T-cell and the target cell is important. The kinetic segregation model for T-cell activation states that the balance of presence of kinases (mainly Lck) versus dephosphorylases (mainly CD45) in the T-cell:antigen-presenting cell interaction zone (immunological synapse) is determinant for TCR downstream signaling and thus for T-cell activation. This balance is mainly influenced by spatial restrictions that appear upon T-cell:target cell interactions.

CD45 enzymes are abundantly present on the membrane of resting T-cells, resulting in sufficient dephosphorylation of the TCR immunoreceptor tyrosine-based activation motifs (ITAMs) to maintain a basal, inactive T-cell status. However, these molecules are relatively bulky and are excluded from the immunological synapse upon TCR-pMHC (peptide major histocompatibility complex) and CD58-CD2 cell adhesion interactions. Consequently, the ITAM phosphorylation equilibrium is outbalanced, and T-cells become activated as a result of downstream intracellular phosphorylation events (52, 53).

This model highlights the importance of the formation of a spatially correct immunological synapse, which is equally relevant in CAR-T cells, as similar signal transduction pathways are determinant for T-cell activation (53). The immunological synapse has been described to have an ideal intermembrane distance of around 15 nm in the center (where TCR-pMHC interactions are mainly taking place) to 100 nm at the edges, where granules and cytokines are exchanged and receptor-ligand pairs are the main interaction molecules (54). As the VHH molecule is anchored to the CAR via a (large) flexible linker, it is difficult to make a concrete estimate of the effective intercellular distance. However, *in silico* modeling of the various VHH-CS1 interactions shows that there are relevant differences between different VHH-CAR:CS1 pairs at this level. These observed differences did not translate directly to differences in CAR-T cell activation potential, and therefore it was assumed that neither location of binding, nor VHH binding orientation are predictive parameters for CAR-T cell activation.

These conclusions are based on sequence-driven computational estimates for the protein structures and interactions. It should be noted here that these are artificial intelligence-based structure interaction predictions, which are not supported by experimental data. Although the AlphaFold2 algorithm has already proven its predictive power for more evolutionary conserved protein interactions (37, 39), it is noteworthy that the program is considered slightly less powerful and therefore less trustworthy for the prediction of antigen-antibody binding. This is because less evolutionary background (on which the modeling is based) is available for these types of interactions (55). As these data do not coincide with the experimental data retrieved from the competition activation assay, one might argue on the accuracy of these modeled interactions. To precisely know the orientation of interaction, experimental data on the interaction structure would be necessary, which could for example be obtained via X-ray crystallography. This is however a technically challenging method that is not recommended to screen complete VHH libraries. All in all, our data demonstrate that the VHH:antigen interaction, as currently computationally modeled, has no predictive value for the VHH-CAR-T cell activation potential. Whether this is due to a lack of accuracy of the prediction or due to a missing link between binding orientation and activation potential, remains an open question.

Within the immunological synapse, the affinity of a TCR for its target pMHC molecule is relatively low, i.e., 1 μM to 100 μM. As such, cells with low target pMHC expression do not trigger T-cell activation and T-cell overstimulation (which may lead to premature exhaustion) is avoided (56). CARs harboring a mAb-derived scFv usually have a (sub)nanomolar affinity for their target (57). This while various studies have shown that an unnecessary increase in affinity of a CAR is more likely to lead to higher toxicity and a shorter T-cell life span (56–58).

Chmielewski and colleagues have identified a ceiling of K_D_ = 10^-8^ M, below which the affinity no longer contributes to enhanced T-cell function (58). It has also been reported that lower affinity CARs are more capable of correctly distinguishing cancerous tumor antigen-overexpressing cells from healthy cells with normal expression levels of a tumor antigen (59). However, this is a balance of sensitivity and selectivity in which (the degree of) antigen expression is also important. Indeed, different studies in different contexts show different results. For example, an optimal CAR affinity in the micromolar range was found for target antigens ICAM-1, CD38 and HER2 (56, 60, 61). Meanwhile, CARs with a nanomolar affinity appeared optimal for tumor antigens EGFR, EGFRvIII and CD123 (62–64). This of course is also related to the avidity of the CAR-antigen interaction, which is subject to not only the affinity, but also to the expression levels of both the CAR on the T-cell surface and the antigen on the cancer cell. Clinical data suggest that the importance of generating an interaction with adequate avidity may prevail over attaining a desired affinity (65).

In any case, these studies highlight the importance of case-by-case fine-tuning of the affinity of a CAR for its target antigen, and the advantage of using the weakest possible affinity binder that is still sufficiently sensitive. Moreover, the balance between selectivity and sensitivity will be even more important in solid tumors, where antigen expression often also occurs on vital healthy tissue. In a hematological context, antigen expression is more likely to be limited to hematopoietic cell lineages, rendering selectivity less critical. All in all, these studies demonstrate that the antigen-binding portion of a CAR has a major impact on its functionality, and that case-by-case selection is needed. When classical scFvs are incorporated, this is less obvious, as they are usually adopted from existing and clinically validated mAbs. Furthermore, the lack of (immune) libraries makes it less straightforward to evaluate multiple scFvs side-by-side.

VHHs have this advantage over scFvs, as they are monomeric by nature and selection usually starts from relatively small immune libraries. Furthermore, VHH-based CARs have already proven their functionality in the clinic, in particular in the context of MM. In the 2022 FDA-approved ciltacabtagene autoleucel (cilta-cel) CAR, antigen-recognition is ensured by two (bi-epitopic) BCMA-specific VHHs (66). This is the second clinically approved CAR-T cell product for the treatment of MM. Idecaptagene vicleucel (ide-cel), also directed against BCMA, was previously approved. In the latter, antigen recognition is provided by a classic scFv format.

Despite the high initial success rates of these therapies, MM relapse remains an important problem, mainly caused by BCMA shedding. These findings demonstrate the power of CAR-T cell therapy for MM, but also its unfulfilled potential due to suboptimal choice of antigen. Therefore, other target antigens are under investigation. Of these, CS1 is of particular interest due to its retained expression after several lines of therapy and its expression pattern that is limited to hematological cell (sub)types. Several CS1-directed CAR-T cell therapies are under investigation, both in a preclinical and clinical stage (11, 67).

Our study combines both optimization points of current CAR-T cell research for MM. In particular, we use a CAR directed against the promising target CS1, and achieve this using a VHH molecule selected out of a library as being optimal in the tested CAR design. Furthermore, we have developed several CS1-specific VHHs that may be complementary to each other. Indeed, antigen expression throughout therapy is an important parameter for its success rate. The combination of therapy and diagnostic imaging is therefore an interesting concept that is increasingly gaining attention in the field of personalized medicine. Since VHHs are ideally suited as radiodiagnostic tools, it would be possible to monitor the status of CS1 expression before and during CS1-targeted (CAR-T cell) therapy. To this end, however, it is important that competition for target binding between the diagnostic and the therapeutic tool is avoided. Thus, compounds that bind a different epitope are needed. Our data lend themselves ideally to such a scenario, as the compounds that perform well *in vivo* do not necessarily correspond to those that provide good CAR-T cell activation, and two different compounds can be optimized according to their intended use.

Finally, it is important to note that although we demonstrate the importance of the incorporated VHH for T-cell activation, this is not the only point of optimization of CAR design and several other factors that may affect CAR-T cell behavior are under investigation (68). These include the other CAR subunits, such as the hinge and transmembrane region (69), as well as the incorporated co-stimulatory molecule(s) (70). The best-known CARs to date are second generation, implying that a single co-stimulation domain is incorporated, mostly 4-1BB or CD28-derived (9, 68). For example, third-generation CARs harbor a combination of two co-stimulatory domains and are currently under clinical evaluation (71). Fourth-generation CARs seek their added value through the local secretion of immune stimulatory cytokines, mainly IL-12 (72).

Another potential influencing parameter that is increasingly gaining attention is the composition and phenotype of the harvested and infused T-cells. As such, the ideal composition of CD4^+^/CD8^+^ T-cell fractions and the added value of eliminating regulatory T-cells are important fields of research (73, 74). Additionally, data show that younger, more stem cell-like phenotypes of T-cells may exhibit superior *in vivo* efficacy, owing to reduced or delayed exhaustion (75, 76). Furthermore, there are many variables in *ex vivo* modification protocols, genetic CAR constructs and manner of cell modification that might influence T-cell behavior (68). In particular, the benefit of reducing CAR-T cell manufacturing time is also gaining interest, as this may not only save patients with rapidly progressing disease and reduce overall costs, but could also potentially be of therapeutic added value, as reduced culturing time might result in a younger (i.e., more stem cell-like) T-cell phenotype (68, 77, 78).

All in all, there are many different variables that determine the behavior and efficacy of CAR-T cells. Our data show that although it has historically received little attention, the antigen-binding component certainly is one of them and that adequate screening is needed.

## 5 Conclusion

Taken together, our results demonstrate that the antigen-binding part of a CAR has a major influence on its functionality, as well as on the activation kinetics of modified CAR-T cells. This emphasizes the importance of selecting, or in the case of scFvs, optimizing this protein domain. We scrutinized several parameters of VHHs trying to link them – without success-, to a superior CAR-T cell response. Consequently, CAR-T cell research remains subject to serendipity and case-by-case selection of the optimal antigen-binding moiety is still needed. Hence, the importance of screening multiple candidate binding domains directly in a CAR configuration cannot be underestimated. In this regard, VHHs have an important advantage over mAb-derived scFvs, due to the easy availability of immune libraries.

## Data availability statement

The original contributions presented in the study are included in the article/**Supplementary Materials**. Further inquiries can be directed to the corresponding author.

## Ethics statement

Ethical approval was not required for the studies on humans in accordance with the local legislation and institutional requirements because only commercially available established cell lines were used. The animal study was approved by ethical committee for use of laboratory animals, Vrije Universiteit Brussel. The study was conducted in accordance with the local legislation and institutional requirements.

## Author contributions

HH: Conceptualization, Data curation, Formal analysis, Funding acquisition, Investigation, Methodology, Project administration, Software, Visualization, Writing – original draft. FM: Data curation, Formal analysis, Investigation, Writing – review & editing. YDV: Conceptualization, Data curation, Formal analysis, Methodology, Writing – review & editing. QL: Conceptualization, Data curation, Methodology, Writing – review & editing. JP: Methodology, Writing – review & editing. PD: Data curation, Investigation, Methodology, Writing – review & editing. TDG: Data curation, Methodology, Software, Writing – review & editing. CG: Investigation, Writing – review & editing. KDV: Conceptualization, Funding acquisition, Methodology, Resources, Supervision, Writing – review & editing. KB: Conceptualization, Funding acquisition, Methodology, Resources, Supervision, Writing – review & editing. ND: Conceptualization, Funding acquisition, Methodology, Resources, Supervision, Writing – review & editing.
