# Supplementary figures and images for: Scrutiny of chimeric antigen receptor activation by the extracellular domain: experience with single domain antibodies targeting multiple myeloma cells highlights the need for case-by-case optimization
Source: Front Immunol. 2024 Apr 19;15:1389018. doi: 10.3389/fimmu.2024.1389018 (PMC11077437; doi:10.3389/fimmu.2024.1389018)

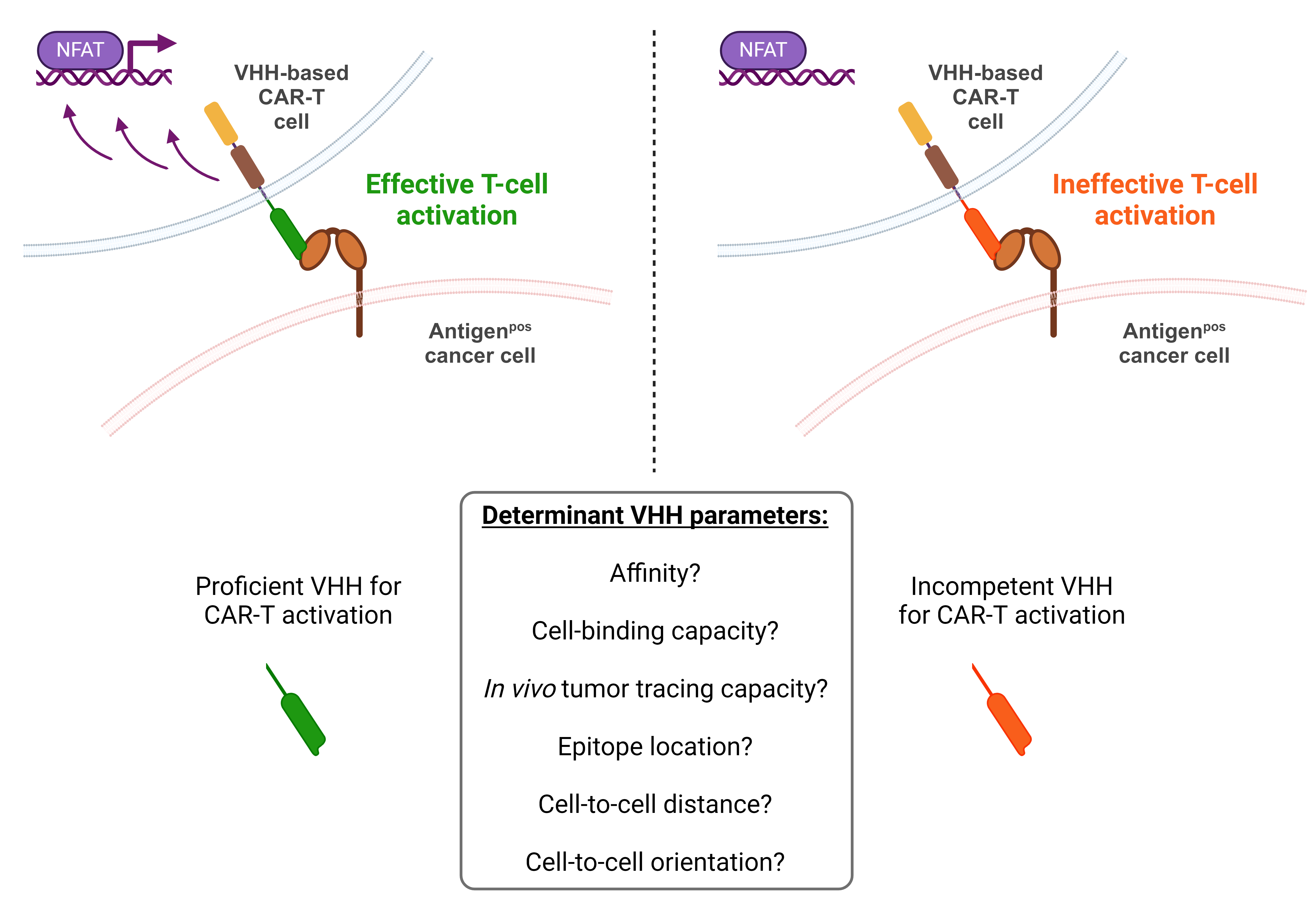

Supplement: Supplementary file 1 [file Image_1.jpeg]
