# Supplementary material for: Scrutiny of chimeric antigen receptor activation by the extracellular domain: experience with single domain antibodies targeting multiple myeloma cells highlights the need for case-by-case optimization
Source: Front Immunol. 2024 Apr 19;15:1389018. doi: 10.3389/fimmu.2024.1389018 (PMC11077437; doi:10.3389/fimmu.2024.1389018)
Supplement: Supplementary file 2 [file DataSheet_1.docx]

Supplementary Material

# Supplementary Figures


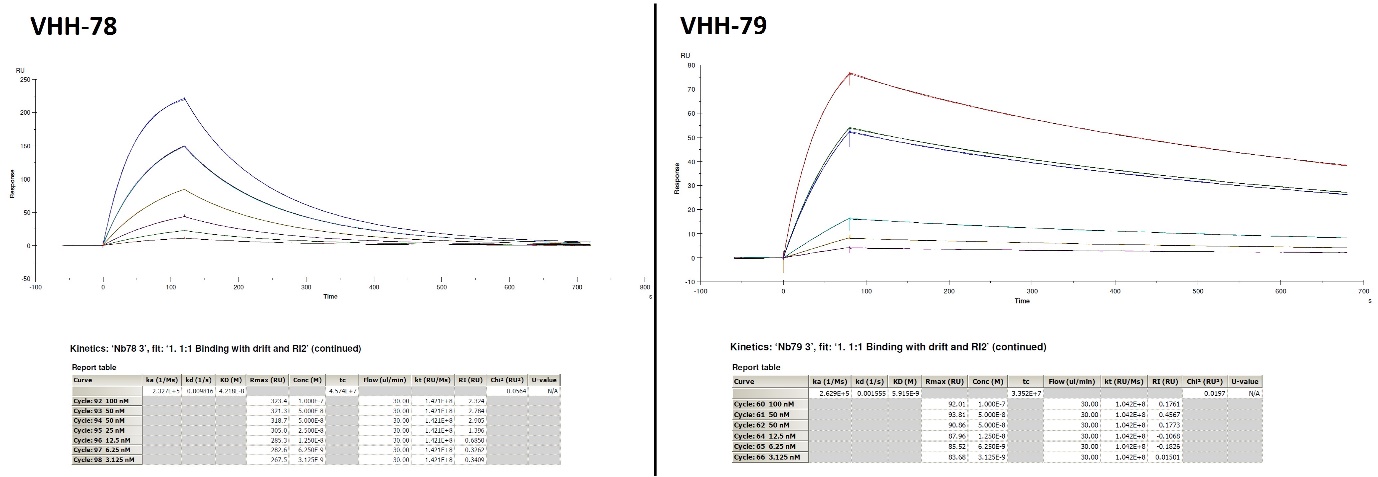


| **k_a_ (M^-1^s^-1^)** | 2.327 x 10^5^ | **k_a_ (M^-1^s^-1^)** | 2.629 x 10^5^ |
| --- | --- | --- | --- |
| **k_d_ (s^-1^)** | 9.816 x 10^-3^ | **k_d_ (s^-1^)** | 1.555 x 10^-3^ |
| **K_D_ (M)** | 4.218 x 10^-8^ | **K_D_ (M)** | 5.915 x 10^-9^ |

**Supplementary figure 1.** ***Affinity determination of VHH-78 and VHH-79 towards murine CS1 in SPR.*** *Sensograms show VHH association and dissociation from a murine CS1-coated CM5 sensor chip in SPR at different VHH concentrations, in a 1/2 dilution series ranging from 100 nM to 3.13 nM with a duplicate measurement at 50 nM. From this, kinetic binding parameters k_a_, k_d_ and K_D_ were calculated, using the ‘1:1 binding with drift and RI2’ fitting model in the Biacore T200 2.0 evaluation Software (GE Healthcare), (n=1).*


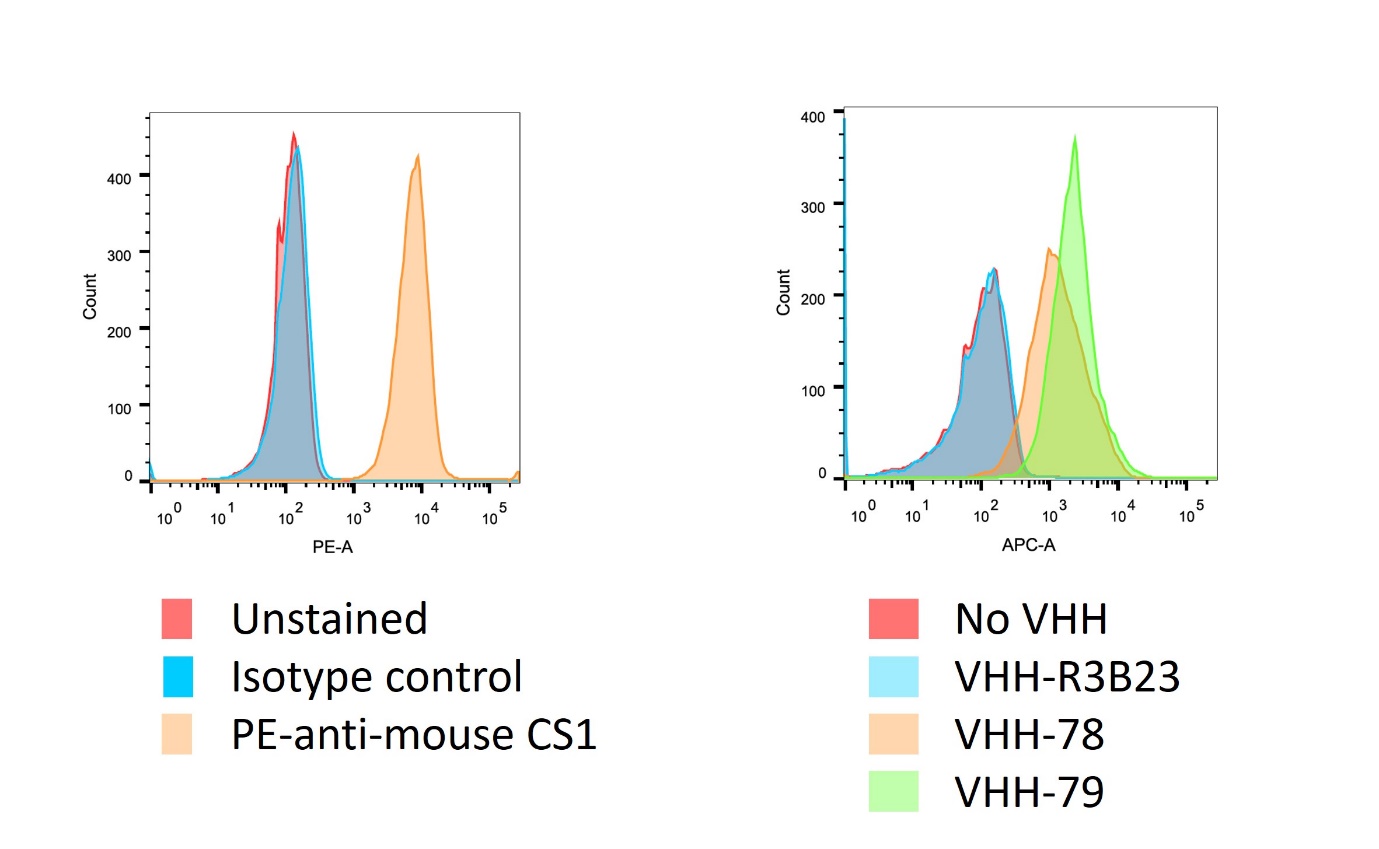


Supplementary figure 2: *Flow cytometry results to confirm VHH-78 and VHH-79 binding to cell-expressed murine CS1 protein. Left: confirmation of antigen expression by 5T33vt target cells, using a PE-labeled anti-mouse CS1 mAb and its isotype control mAb (Biolegend). Right: Cell binding of the murine CS1-specific VHHs 78 and 79 is confirmed after VHH incubation at 200 nM VHH on murine CS1^pos^ 5T33vt MM cells, followed by staining for the C-terminal VHH hexahistidine tag using a primary mouse anti-His IgG1 mAb (Biolegend) and a secondary allophycocyanin (APC)-labeled anti-mouse IgG1 mAb (Biolegend). The situation in which the non-targeting control VHH R3B23 (200 nM) is added is shown by the blue histogram and the one in which no VHH was added to the cells is displayed by the red histogram (n=1).*


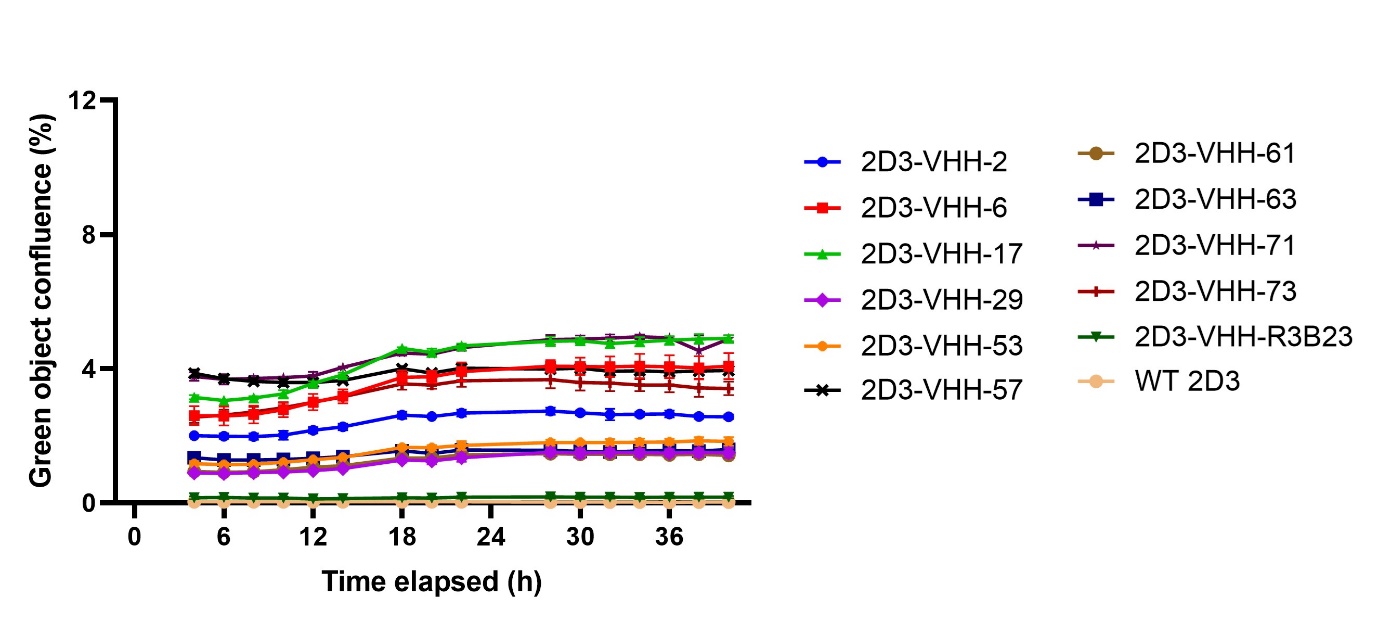


Supplementary figure 3: *Real-time follow-up of green fluorescence in the IncuCyte Zoom Live-Cell Analysis System of co-cultures of CS1^neg^ JJN3 MM cells with the different CS1-specific 2D3-VHH cell lines, the negative control 2D3-VHH-R3B23 and the wild type (WT) 2D3 cell line at a (1:10) cell ratio. Data are quantified as mean green object confluence (%) ± SD (n=3).*
